# Neo-taphonomic analysis of the Misiam leopard lair from Olduvai Gorge (Tanzania): understanding leopard–hyena interactions in open settings

**DOI:** 10.1098/rsos.220252

**Published:** 2022-07-20

**Authors:** Manuel Domínguez-Rodrigo, Elia Organista, Enrique Baquedano, Gabriel Cifuentes-Alcobendas, Marcos Pizarro-Monzo, Marina Vegara-Riquelme, Agness Gidna, David Uribelarrea, David Martín-Perea

**Affiliations:** ^1^ Institute of Evolution in Africa (IDEA), Alcalá University and Archaeological and Paleontological Museum of the Community of Madrid, Covarrubias 36, 28010 Madrid, Spain; ^2^ Area of Prehistory (Department History and Philosophy), University of Alcalá, 28801 Alcalá de Henares, Spain; ^3^ Department of Anthropology, Rice University, 6100 Main Street, Houston, 77005-1827 TX, USA; ^4^ Osteoarchaeological Research Laboratory, Department of Archaeology and Classical Studies, Stockholm University, Wallenberglaboratoriet, SE-106 91, Stockholm, Sweden; ^5^ Archaeological and Paleontological Museum of the Community of Madrid, Plaza de las Bernardas s/n, 28801 Alcalá de Henares, Spain; ^6^ Cultural Heritage Department, Ngorongoro Conservation Area Authority, PO Box 1, Ngorongoro Crater, Arusha; ^7^ Geodynamics, Stratigraphy and Palaeontology Department, Complutense University of Madrid, José Antonio Novais 12, 28040, Madrid, Spain; ^8^ Paleobiology Department, National Natural Sciences Museum – CSIC, José Gutiérrez Abascal 2, 28006, Madrid, Spain

**Keywords:** leopard, Olduvai Gorge (Tanzania), hyena, taphonomy, ethology

## Abstract

Misiam is a modern wildebeest-dominated accumulation situated in a steep ravine covered with dense vegetation at Olduvai Gorge (Tanzania). It is interpreted here as a leopard lair to which carcasses have been transported for several years. Felid-specific bone damage patterns, felid-typical skeletal part profiles, taxonomic specialization and the physical presence of leopards observed by the authors show that leopards at Misiam can be specialized medium-sized carcass accumulators. Hyenas also intervened at intervals in the modification of the retrieved faunal assemblage. This makes Misiam a carnivore palimpsest. Here, we additionally show that leopards only transport and accumulate carcasses on occasions, that they can seem highly specialized despite being dietary generalists, and that such a behaviour may be prompted by seasonal competition or during the breeding season or both. Misiam is the first open-air leopard lair with a dense bone accumulation reported. There, leopards engaged in intensive accumulation of carcasses during the wet season, when the southern Serengeti short-grass plains undergo the effect of the famous wildebeest migration and this migratory species reaches the gorge. The ecological importance of this behaviour and its relevance as a proxy for reconstructing prehistoric carnivore behaviours are discussed.

## 1. Introduction

Leopards have been documented as the only African felid that can systematically accumulate carcasses in their lairs or denning areas [1]. Exceptionally, lions have been interpreted as capable of accumulating carcasses, under situations of socio-ecological stress, nomadic behaviour, and on a seasonal basis, taking advantage of ecosystemically localized episodes or herbivore mass migrations [2,3]. Out of the felid guild, leopards have been more frequently palaeoanthropologically invoked, especially for their role as predators of hominins or as sources of food for kleptoparasitic hominins (e.g. [1,4]); however, they still constitute an understudied taphonomic agent. Simons [5] reported on leopard-deposited carcasses in the Mount Suswa lava tube caves in Kenya. Sutcliffe [6] documented leopard occupation of two caves in Mount Elgon in Kenya. There, he documented remains of eight or nine leopards and up to 37 baboon carcasses. He was the first one to document the intense deletion of the axial skeleton (i.e. vertebrae and ribs) of baboons after leopard consumption. Brain [1] carried out actualistic observations on cheetah-fed ungulate and baboon carcasses, and he also studied several natural leopard lairs. In addition, he documented live destruction of leopards on their prey at Kruger National Park. There, he also documented that the axial skeleton of small ungulates gets virtually destroyed during consumption. More recently, Cavallo [7] studied leopard carcass-caching behaviour and its taphonomic consequences in the Serengeti (Tanzania). Pickering and colleagues conducted experiments feeding baboon carcasses to captive leopards in South Africa and also collected baboon carcasses consumed by wild leopards [8–10]. These authors produced the most detailed description of taphonomic modifications underwent by baboons when consumed by leopards. In general, these studies have reported in detail bone damage patterns in small carcasses consumed by leopards. To this, the most recent addition is the study of the baboon carcass accumulation of Misgrot Cave (South Africa) [11]. However, leopards do not only consume small animals. De Ruiter & Berger [12] also documented leopards accumulating medium-sized carcasses in dolomitic caves. Bone damage on those larger carcasses is less intense, but conspicuous nonetheless.

An analytic comparison between spotted hyenas and leopards showed that both agents are quite diagnostic and taphonomically differentiable, despite the high variability of their behaviour (especially in hyenas) [13,14]. Comparative research demonstrates that the pattern of bone modification resulting from leopard carcass consumption is similar to that of lions (*Panthera leo*) and cheetahs (*Acinonyx jubatus*) [13,15,16]. Thus, one might argue that there exists a diagnostic ‘felid taphonomic signature’. This is confirmed when using long bone taphotype analyses [17]. Taphotypes elaborated on furrowing patterns on long bone epiphyseal ends show statistical differentiation between felids and hyenids [17]. Nevertheless, in most published research on wild leopard accumulations in cave or rock shelter lairs, there surely is intrusion of other taphonomic agents, such as hyenas, jackals, foxes or porcupines. ‘Open-air’ accumulations of carcasses by leopards have only been restricted to the redundant use of the same locus for tree-stored carcasses [7]. However, interpretations of some early Pleistocene sites as palimpsests of felid-accumulated and hyena-modified faunal assemblages, such as the 1.8 Ma site of FLK North at Olduvai Gorge [13,15,16]), require a better understanding of felids, and more specifically leopards, as taphonomic agents.

Leopards are secretive predators, whose study in the wild is difficult because of their elusive character. However, their carcass consumption behaviour is tightly linked to that of other competitors, namely hyenas. In areas of competition, leopards transport their prey to their lairs, denning areas, or to trees [7]. Open-air lairs are common and occur in dense vegetation zones. One of those zones occurs in the southern Serengeti ecosystem, in the vicinity of the short-grass plains occupied during the wet season by the great wildebeest migration. These plains reach the rim of the palaeoanthropologically famous Olduvai Gorge. The vertical and sloping walls of the gorge are covered by discontinuous patches of dense thicket and forest. Over the years, in the immediacy of some of these patches, we have witnessed abundant leopard footprints. Hyenas are also active in the vicinity of these areas. Sometimes, we have witnessed the alternation in the use of some of these patches by leopards and hyenas. Leopards use the dense vegetation to create a series of ‘tunnels’ or paths, within which they stay away from other predators. They frequently bring their prey there to be eaten safely, since trees in the surrounding area are too low for them to carry their prey up to branches. Their strategy is to ambush prey in the overlying plains and then transport it to the safety of the lairs inside the gorge.

**Figure 1.**
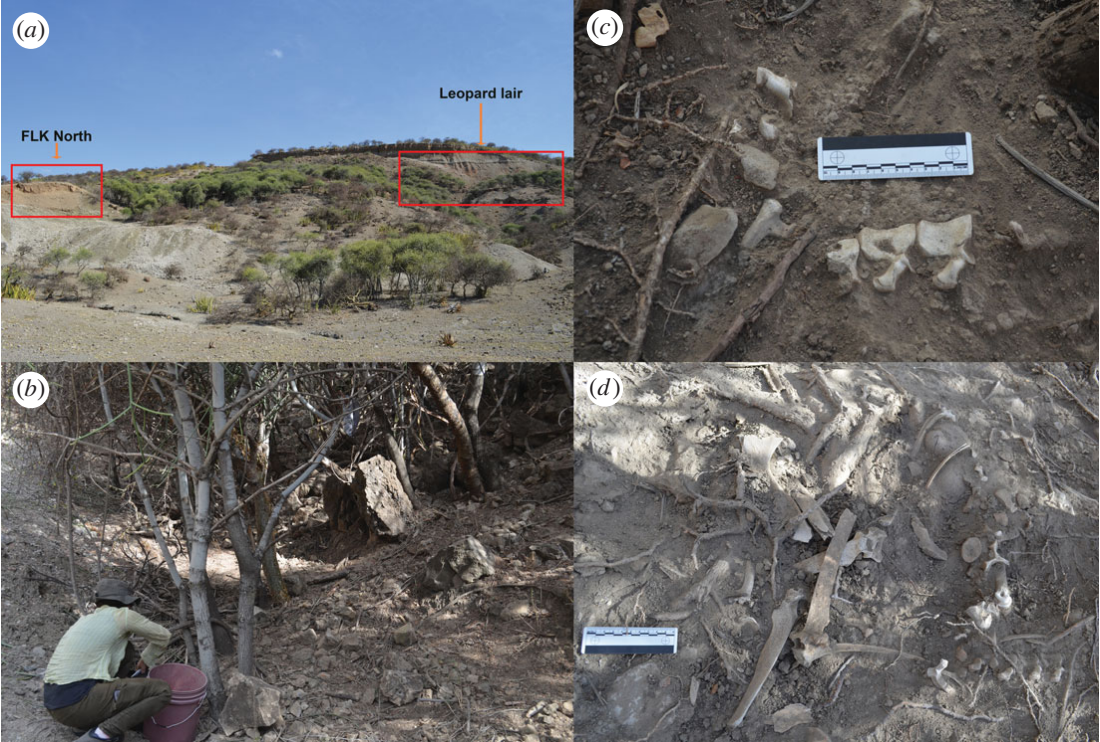
(*a*) Location of the dense vegetation patch used by leopards as a lair. (*b*) Slope aspect of the lair cleared of vegetation. Bones occurred along the slope. Articulated elements (*c*) and mixed elements from several carcasses (*d*) deposited on the same spot.

Here, we will report on one of these densely vegetated patches used as a leopard lair and den. The lair occurs in a ravine, named locally by the Maasai as Misiam, which extends above the famous site of FLK North [18]. In 2016, the local Maasai cleared the ground vegetation, leaving only the short trees. This enabled us to ‘enter’ the lair and collect a large collection of bones that laid on the surface. The lair is on a highly tilted slope, which over the years produced bone movement by gravitation (figure 1). The slope debris covered a substantial part of the area and a small test trench showed that there are large amounts of bones still covered by modern soil. Since 2016, we have been monitoring the lair and have found evidence of use by leopards by the recurrent presence of their footprints. This was definitely confirmed in 2022, when both an adult and a cub were found inside a small pipe caused by erosion of the Bed III sediments.

Hyenas, namely striped hyenas, have also been seen active near the lair. A recent experiment carried out about 300 m away from the lair showed the presence of a striped hyena during the night [19]. In a second leopard lair, we documented the alternation in the use of the space by leopards and hyenas; in both cases, both agents used the spot as a denning place. At Misiam, this has not been documented. However, we do believe that in the intervals in which leopards were not actively using the lair, striped and spotted hyenas may have used it briefly, which would be reflected on some degree of long bone breakage documented on medium-sized carcasses. This is not typical of leopards, but it is highly diagnostic of hyenas. Bone damage by hyenas at Misiam is limited, probably because when they accessed the site, bones were already getting dry. We also believe that leopards feel safe in their densely vegetated lairs because several of the hyenas that we have seen active inside the gorge are striped hyenas, which are of solitary habits, compared with spotted hyenas, and do not constitute a threat to leopards. However, Misiam, like probably most other leopard lairs inside Olduvai Gorge, constitutes a good example of palimpsestic use of the same spaces by felids and hyenids, with variable taphonomic imprints according to the type of interaction and usage of the same loci by both predators. Here, we will analyse the surface collected bone assemblage and will show that leopard bone damage is quite diagnostic. We will also show that when not pressed to store food in trees, leopards are successful hunters of medium-sized carcasses, as already suggested by the accumulation of large carcasses at WU-BA 001 (South Africa) [12]. We will also compare the Misiam assemblage with other classical leopard faunal assemblages to analyse the extent of the variability in their carcass-processing behaviour.

## 2. Methods

### 2.1. Skeletal profiling

In the present study, the standard zooarchaeological units to determine skeletal part abundance have been used: the number of identifiable specimens (NISP), the minimum number of elements (MNE), the minimum number of individuals (MNI), the minimum number of animal units (MAU) and their relative frequency (%MAU) [20]. In Bunn's [21] method, small carcasses refers to sizes 1 and 2 (up to 120 kg in live weight), medium-sized refers to size 3 (ranging from 120 to 450 kg), and large refers to sizes 4 through 6 (exceeding 450 kg). Here, we will use medium-sized, because the largest animal (wildebeest) identified in the Misiam assemblage is a typical size 3 carcass size. Long limb bones were also divided into upper (humerus and femur), intermediate (radius-ulna and tibia), and lower (metacarpal and metatarsal) long limb bones [22]. These, in turn, have been divided into proximal and distal epiphyses, proximal shaft, midshaft and distal shaft. The MNE estimates were based on the systematic inclusion of shaft specimens using a manual overlap approach [23], and taking into account the size, side, landmarks and ontogenetic age of each identifiable specimen [24]. Based on carnivore preferential bone element and portion destruction, and given the strong impact that carnivore-ravaged assemblages have on the biasing of original skeletal part profiles, Marean & Cleghorn [25] have differentiated between high and low survival elements. High-survival elements are those that contain enough dense cortical bone to withstand destruction processes (namely, cranio-mandibular elements and long limb bones). Low-survival elements are those composed mainly of cancellous or trabecular bone, such as axial (i.e. vertebrae and ribs), some appendicular elements (e.g. compact carpo-tarsal and phalangeal bones), and coxal and scapular elements [26].

### 2.2. Bone breakage and element modification

Bone damage will be described per element. In this section, bone damage refers to green bone breakage [27,28], caused by either gnawing or furrowing. After a thorough description per anatomical section, bone breakage of long bones will be specifically addressed. Here, we will consider shaft circumference types [21], bone breakage planes (oblique, longitudinal and transverse) [28] and notch types. Fracture notches were identified according to the typological classification proposed by Pickering & Egeland [29] (modified from Capaldo and Blumenschine [30]: (i) complete notches (Type A) have two inflection points on the cortical surfaces and a non-overlapping negative flake scar; (ii) incomplete notches (Type B) are missing one of the inflection points; (iii) double overlapping notches (Type C) have negative flake scars that overlap with an adjacent notch; (iv) double opposing complete notches (type D) are two notches that appear on opposite sides of a fragment and result from two opposing loading points; and (v) micro-notches (Type E, less than 1 cm). The long bone circumference shaft analysis will use different assemblages for comparison: the KND2 [31] and Syokimau [32] hyena dens, a captive spotted hyena assemblage [33] and three human assemblages; two modern (hammerstone-only, Khwee camp) [32,33] and one archaeological (Sonai) [31].

### 2.3. Bone surface modifications (BSM)

Cortical surface preservation was evaluated by taking into consideration the stages of subaerial exposure [34]. Cortical surfaces were examined with 10×–40× hand lenses under a strong oblique light source. Several types of marks were identified: tooth-marks, trampling and biochemical marks. The analysis applies a ‘configurational approach’ where mark morphology, the anatomical placement of marks, and the sedimentary context of the specimen are taken into consideration [35]. The identification of tooth-marks was made following the criteria outlined by Blumenschine [36]. The identification of trampling followed the guidelines described by Domínguez-Rodrigo *et al.* [37]. Biochemical marks or bioerosive modifications followed the criteria described by Domínguez-Rodrigo & Barba [38,39].

### 2.4. Subaerial weathering

In order to understand deposition time, weathering stages of bones were tallied per element, following Behrensmeyer 6-stage criteria [34]: stage 0 implies the absence of cracking; stage 1 involves some cracking parallel to the fibre structure; stage 2 displays some flaking associated with cracking of the external cortical layer; stage 3 is characterized by areas of weathered compact bone with fibrous texture, with the outer cortical layer disappeared; stage 4 is broadly fibrous, with most of the external cortical layer and part of the internal fibrous tissue flaked off; and stage 5 consists of fibrous bone texture in a bone matrix that is falling apart [34].

### 2.5. Comparative analysis

In order to compare skeletal profiles in felid and hyenid assemblages, we will use some of the most representative assemblages in the literature. For spotted hyena dens, we will use data from the Koobi Fora Hyena Den 1 (KFHD1) [40], the Amboseli den [41,42], the Maasai Mara den [43] and the Syokimau den [44], all of them in Kenya, and the Eyasi (Kisima Ngeda) Hyena Den 2 (KND2) (Tanzania) [31]. We use these assemblages also because they are either dominated by size 3 carcasses or these make up a significant part of the assemblage. In KFHD1, crocodile remains make up 35% of the assemblage [40]. In KND2, medium-sized animals comprise 33% of the assemblage [31]. KFHD1 and KND2 will also be used as controls for patterns in small-sized fauna.

When comparing long bone shaft breakage patterns, we also used additional hyena-made assemblages: Dumali, Heraide, Yangula Ari, Oboley (spotted hyenas), Datagabou (striped hyena, Djibouti) and Uniab (brown hyena, Namibia) [45]. These assemblages are almost completely dominated by very small fauna (*Capra hircus*), and several of them constitute significantly smaller sample sizes than the hyena dens mentioned above; only Dumali and Uniab have more than 400 specimens, and some (e.g. Yangula Ari) have as few as 210 specimens. This is why they were not included in the skeletal profile analysis, since in order to characterize agency in the Misiam assemblage, only larger samples including a more prominent representation of larger fauna were included.

The leopard lairs used for comparison are: Portsmut and Hakos River (Namibia) [1], and WU/BA-001 (South Africa) [12]. Portsmut and Hakos River show a low density of remains, probably also modified by porcupines or other agents. The remains belonging to larger animals show an interesting contrast with those documented in hyena dens: the presence of axial and compact bones is high. These latter bones are also well represented in smaller carcasses. This characteristic is more marked in WU/BA-001 [12]; the least altered leopard lair documented to date. This lair was monitored for 7 years. These samples were initially used for comparative analysis by Domínguez-Rodrigo and Pickering [46].

All the comparative assemblages were transformed into %MAU to account for differential inter-assemblage quantitative representation [47,48]. First, they were analysed using generalized low rank models (GLRM) as an exploratory method. Then, we used a uniform manifold approximation and projection (UMAP), to classify leopards' and hyenas' bone assemblages, especially according to each feature. Lastly, we used a cluster analysis with variance-dependent phylogenetic tree to show the actual distances among all the assemblages compared.

GLRM are a series of methods for dimensionality reduction that use several loss function types and can implement regularization functions. Whereas principal component analysis (PCA) is based on orthogonal projections of linear relationships, in cases where relationships are nonlinear, the PCA underperforms compared with other more flexible methods. GLRM decomposes a table into two distinctive matrices X and Y. X contains the same number of rows as the original table, but all variables are condensed into k factors. Y has k rows and the same number of columns as features (i.e. variables) in the original table. Each of the rows is an archetypal feature derived from the columns (i.e. variables) of the original table. Each row of X corresponds to a row of the original table projected into this reduced dimension feature space. Data are compressed by the low-rank representation derived from k feature reduction. An advantage of GLRM over PCA is that it can handle mixed datasets containing numeric, categorical and Boolean data. GLRM admits several types of loss functions: Huber, Poisson, quadratic, periodic or hinge. It also allows the use of regularization functions, including: Lasso, Ridge, OneSparse, Simplex, UnitOneSparse and quadratic. Loss functions are used to select the optimal archetypal values. Regularization is used to limit X and Y archetypal values. This impacts the effect of negative data, multicollinearity and overfitting. In the present analysis, GLRM was performed with the ‘h2o’ R library (www.r-project.org).

UMAPs is a nonlinear dimension-reduction method based on finding inter-case distances in a low-dimensional feature space. The key of UMAP over other dimension-reduction nonlinear methods, like t-distributed stochastic neighbour embedding (t-SNE), is that distances are generated along a ‘manifold’. A manifold is an n-dimensional geometric shape constituted of the path(s) among the points. Every point is referenced according to a small two-dimensional neighbourhood around it. The UMAP algorithm searches for a multi-dimensional space delimited by the location of points. UMAP uses a nearest-neighbor approach, by eventually connecting all the points along its search regions. This forces a uniform distribution of points. The distances of points along this manifold are then derived through Euclidean distances. Several optimization methods can be used to reproduce inter-point distances. For the latter process, the UMAP approach that we will use is based on a cross-entropy loss function. For the UMAP analysis, we have used the ‘umap’ R library (www.r-project.org). We have also used a search grid combining ranges of values for number of neighbors, minimal distance between neighbors, distance metric, and number of epochs (i.e. iterations of the optimization process).

Finally, a hierarchical cluster analysis, using a Euclidean distance matrix on the %MAU dataset, was carried out. The method used was the ‘average’ linkage, which represents the average distance between the points. The combination of the three methods was used to study agent-specific variability in inter-assemblage element representation.

### 2.6. Orientation patterns

**Figure 2.**
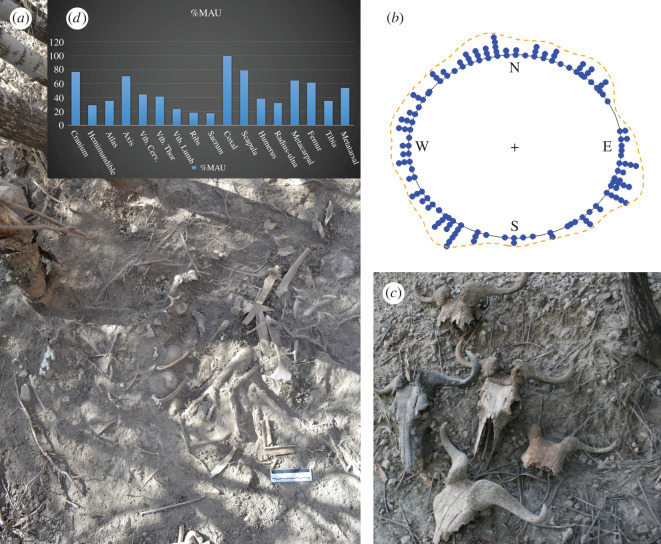
(*a*) Area selected for shallow excavation and full of bones of several carcasses. (*b*) Dot diagram showing the orientation distribution of the bones collected in the test pit. The diagram is accompanied by a kernel density line. (*c*) Examples of wildebeest skulls found in the lair. (*d*) %MAU distribution according to skeletal element.

Given the location of the assemblage on a tilted slope, the scattering of bones over a large surface of the slope must have been affected by rains, trampling and gravity. This could be reflected on potential anisotropic patterns of bone orientation. For this purpose, a small area on the slope was selected and a small trench (2 × 1 m) was excavated to a depth of 25 cm. The assemblage retrieved from this unit was treated as a sample of the rest of the assemblage for orientation data (figure 2). Circular data resulting from the analysis of this trench were statistically treated by using R. Data were originally obtained in degrees. Circular objects were then analysed using the ‘circular’ R library. Isotropy (or randomness in orientation) can be statistically assessed by using tests against unimodal distributions, and omnibus tests against bimodal and multimodal distributions. To test uniform distributions against unimodal distributions, the Rayleigh's (R) test was applied (Fisher, [49]). To analyse multimodal distributions, the Kuiper's test and the Watson's test (V) were used.

A typical model for assessing the normal distribution of circular data is the von Mises distribution. For this distribution, the dispersion is quantified by a concentration parameter k, with k = 0 corresponding to an isotropic distribution and increasing values with a trend towards anisotropy. The Watson (U^2^) test is a goodness-of-fit statistic for the von Mises distribution and is recommended as a general test for uniformity. Values of the omnibus tests with *p* > 0.05 indicate that the null hypothesis of isotropy cannot be rejected. The three tests were applied in the present study and the R functions used were ‘rayleigh.test’, ‘kuiper.test’ and ‘watson.test’ from the R ‘circular’ library. Results were double-checked against the same tests from the ‘CircStats’ R library. A dot diagram with a kernel density line was used to plot the results. Kernel density estimates were made testing different band widths and selecting the optimal value. The final solution adopted a band width of 75. The sample of bones collected in the test pit was 144 specimens.

## 3. Results

### 3.1. Skeletal profiling

The Misiam faunal assemblage is composed of a minimum of 25 individuals (two fetal individuals and two juveniles; the remainder are adults). There are 23 medium-sized bovids, as inferred from the left coxal bone. Most of them can be identified as wildebeests. All bovid dental remains are also identified as wildebeests. In addition, there is an equid and a gazelle (*Gazella thompsonii*).

Table 1 shows the anatomical profile of the Misiam bone assemblage. Tables 2 and 3 show the element, MAU and %MAU distribution for each of the comparative hyena and leopard samples analysed. The three comparative analyses showed that Misiam was clearly a leopard-made faunal accumulation, with a substantial impact by hyenas. This will be seen in more detail in the bone damage sections below.

**Table 1.** Number of identifiable specimens (NISP), minimum number of elements (MNE), minimum number of animal units (MAU) and relative MAU (%MAU) of the Misiam assemblage.

	NISP	MNE	MAU	%MAU
cranium	16	13	13	76.40
hemimandible	10	10	5	29.41
atlas	6	6	6	35.29
axis	12	12	12	70.59
cervical vert	38	38	7.6	44.71
thoracic vert.	92	92	7.1	41.76
lumb vert.	25	25	4.1	24.12
ribs	132	85	3.2	18.82
sacrum	3	3	3	17.65
pelvis	39	34	17	100
scapula	28	27	13.5	79.41
humerus	24	13	6.5	38.24
radius-ulna	27	11	5.5	32.35
metacarpal	28	22	11	64.71
femur	28	21	10.5	61.76
tibia	35	12	6	35.29
metatarsal	21	18	9	52.94
compact bones	5	5	0.17	1.000

**Table 2.** Minimum number of elements (MNE), minimum number of animal units (MAU) and relative MAU (%MAU) in each of the hyena dens used: Amboseli (wildebeest), Maasai Mara carcass sizes 3 & 4 (MM-3&4), Maasai Mara carcass size 2 (MM-2), Syokimau carcass sizes 1 and 2 (Syok-1&2) and 3 and 4 (Syok-3&4), and Kisima Ngeda hyena den 2 (KND2) on small carcasses (ovicaprids). Data for the hyena dens were taken from Kerbis [50] for Amboseli and Maasai Mara, Bunn [22] for Syokinau and Prendergast & Dominguez-Rodrigo [31] for KND2.

	Amboseli-3	MAU	%MAU	MM-3&4	MAU	%MAU	MM-2	MAU	%MAU	Syok-1&2	MAU	%MAU	Syok-3&4	MAU	%MAU	KND2	MAU	%MAU
mandible	6	3	33.3	24	12	60	20	10	100	7	3.5	100	10	5	62.5	20	10	66.6
cranium	9	9	100	20	20	100	10	10	100	3	3	85.7	8	8	100	15	15	100
vertebrae	11	0.42	0.04	10	0.38	1.9	3	0.11	1.1	3	0.11	3.1	4	0.15	1.8	33	1.26	8.4
scapula	4	2	22.2	11	5.5	27.5	4	2	20	2	1	28.5	3	1.5	18.7	8	4	26.6
humerus	11	5.5	61.1	17	8.5	42.5	4	2	20	2	1	28.5	11	5.5	68.7	11	5.5	36.6
radius-ulna	9	4.5	50	23	11.5	57.5	5	2.5	25	1	0.5	14.2	6	3	37.5	10	5	33.3
metacarpal	7	3.5	38.8	14	7	35	3	1.5	15	2	1	28.5	4	2	25	2	1	6.6
pelvis	3	1.5	16.6	6	3	15	2	1	10	3	1.5	42.8	2	1	12.5	7	3.5	23.3
femur	3	1.5	16.6	9	4.5	22.5	2	1	10	0	0	0	2	1	12.5	9	4.5	30
tibia	7	3.5	38.8	22	11	55	5	2.5	25	2	1	28.5	5	2.5	31.2	27	13.5	90
metatarsal	11	5.5	61.1	11	5.5	27.5	3	1.5	15	2	1	28.5	3	1.5	18.7	7	3.5	23.3
compact bones	26	1.08	12	54	2.25	11.2	9	0.34	3.4	4	0.15	4.2	9	0.34	4.2	10	0.38	2.5
ribs	1	0.03	0.33	1	0.03	0.15	0	0	0	1	0.03	0.85	1	0.03	0.37	24	0.92	6.1
TOTAL	108			222			70			32			68			183

**Table 3.** Minimum number of elements (MNE), minimum number of animal units (MAU) and relative MAU (%MAU) in each of the leopard-accumulated assemblages used: Portsmut, Hakos, WU-BA001, and Misiam. Data for Portsmut and Hakos were taken from Brain [1] and those for WU-BA001 from de Ruiter and Berger [51].

	Portsmut leopard lair			Hakos River leopard lair						WU/BA-001						Misiam		
	small carcasses			small carcasses			large carcasses			small carcasses			large carcasses			large carcasses		
	MNE	MAU	%MAU	MNE	MAU	%MAU	MNE	MAU	%MAU	MNE	MAU	%MAU	MNE	MAU	%MAU	MNE	MAU	%MAU
mandible	2	1	33.3	3	1.5	50	3	1.5	42.8	4	2	50	9	4.5	65.2	10	5	29.4
cranium	3	3	100	3	3	100	3	3	85.7	3	1.5	37.5	5	5	72.4	13	13	76.4
vertebrae	8	0.3	10	1	0.5	16.6	17	0.65	18.5	47	1.8	45	60	2.3	33.3	173	6.65	39.2
scapula	2	1	33.3	2	1	33.3	1	0.5	14.2	5	2.5	62.5	8	4	57.9	28	14	82.3
humerus	0	0	0	1	0.5	16.6	7	3.5	100	5	2.5	62.5	8	4	57.9	13	6.5	38.2
radius-ulna	1	0.5	16.6	1	0.5	16.6	4	2	57.1	5	2.5	62.5	8	4	57.9	11	5.5	32.3
metacarpal	1	0.5	16.6	0	0	0	3	1.5	42.8	5	2.5	62.5	8	4	57.9	22	11	64.7
pelvis	1	0.5	16.6	0	0	0	2	1	28.5	2	1	25	8	4	57.9	34	17	100
femur	1	0.5	16.6	0	0	0	6	3	85.7	5	2.5	62.5	9	4.5	65.2	21	10.5	61.7
tibia	1	0.5	16.6	1	0.5	16.6	7	3.5	100	4	2	50	10	5	72.4	12	6	35.2
metatarsal	2	1	33.3	0	0	0	4	2	57.1	4	2	50	10	5	72.4	18	9	52.9
compact bones	25	0.96	32	6	0.23	7.6	45	1.73	49.4	104	4	100	180	6.9	100	5	0.17	1
ribs	0	0	0	0	0	0	3	0.11	3.1	26	1	25	114	4.38	62.3	85	3.2	18.8
TOTAL	47			18			105			219			437			445		

Misiam is characterized by an anatomical representation of all elements, with a good presence of the high-survival element set (cranio-mandibular and long limb bones), as well as the low-survival axial, pelvic and scapular set. The most under-represented elements are ribs, and compact bones (carpo-tarsal and phalanges) (table 2; figure 2). This could be the result of rib destruction during carcass consumption and compact bone selective recovery, given that the assemblage was not excavated, but collected from the surface. All this shows that carcasses were transported into the Misiam lair in a fairly complete state.

**Figure 3.**
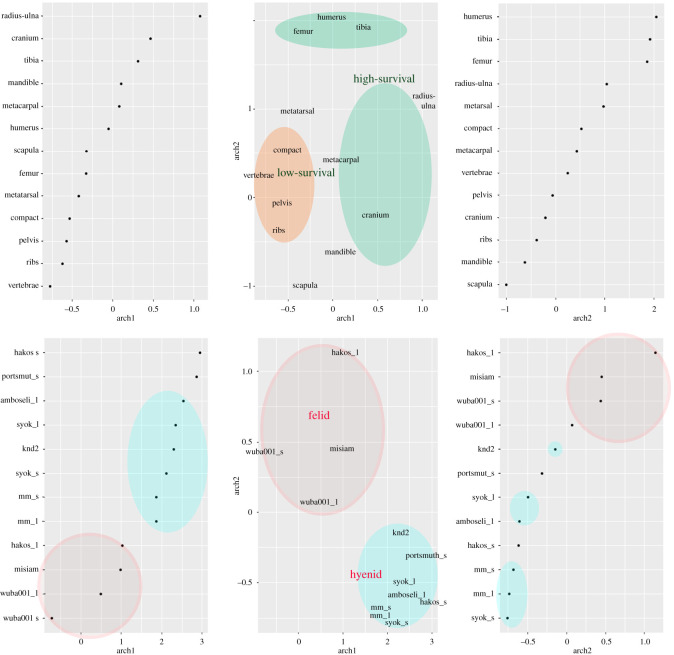
LRGM analysis of the skeletal parts (above) and faunal assemblages (below), showing their respective importance in each archetype (sides) and their distribution in the Euclidean space. Ovals show delimited areas of agent-specific concentration variance. The occurrence of each hyena and leopard assemblage appear thus spatially referenced regarding the distribution of each element representation. s, small; l, larger (medium-sized); syok, Syokimau; mm, Maasai Mara; knd, Kisima Ngeda.

An iterative process of GLRM with a hyperparameter search grid combining different types of regularization and gamma values from 0 to 1, yielded an optimal basic model (involving no regularization and gamma = 0) with error less than 0.005 and quadratic loss. This is expected given the exploratory use of GLRM here instead of targeting classification of larger datasets. The procedure used *k* = 10 and the solution yielded a model with the two main archetypes accounting for 72% of the sample variance (archetype 1 = 54.44%; archetype 2 = 17.57%). The first archetype separated neatly the high-survival (positive values) from the low-survival (negative values) bone elements (figure 3). The second archetype separated the long bones from the cranial and mandibular elements. Regarding the low-survival axial elements, the second archetype also separated the cubic-shaped elements (compact bones) from the flat bones (ribs and scapulae). When projecting the carnivore assemblages in this space, two clear clusters emerge. One contains all the hyena dens regardless of carcass size. It also contains two leopard samples from small animals. This indicates that leopard bone damage on small carcasses can be very similar to that documented in hyena dens both on small and large carcasses [14]. However, leopard damage of medium-sized carcasses is distinctively different. This is shown in the second cluster of leopard assemblages composed of larger animals. The presence of WU-BA001 sub-assemblage of small carcasses in this second cluster, which exhibited minimal damage by carnivores other than leopards [12], also underscores the probability that Portsmut and Hakos may have experienced some damage by other agents. The central position of the leopard assemblages in this second cluster is explained by the balanced preservation of low- and high-survival elements. In this dual clustering, the Misiam assemblage clearly appears associated with the leopard cluster. The GLRM classifies it clearly as a leopard assemblage.

**Figure 4.**
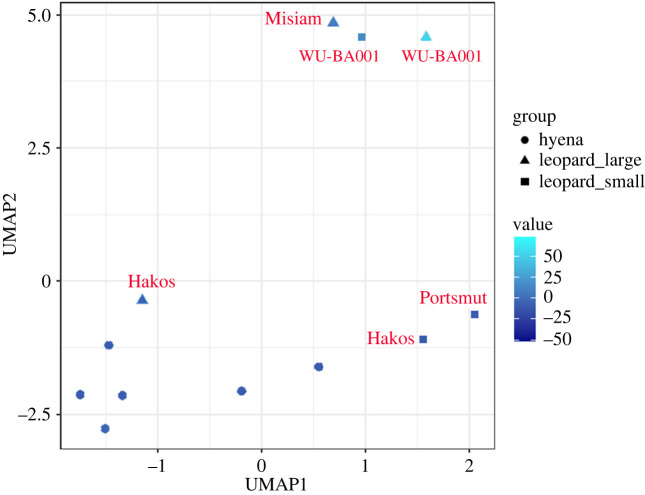
UMAP two-dimensional solution of leopard- and hyena-made bone assemblages. Only the leopard assemblages appear labelled.

**Figure 5.**
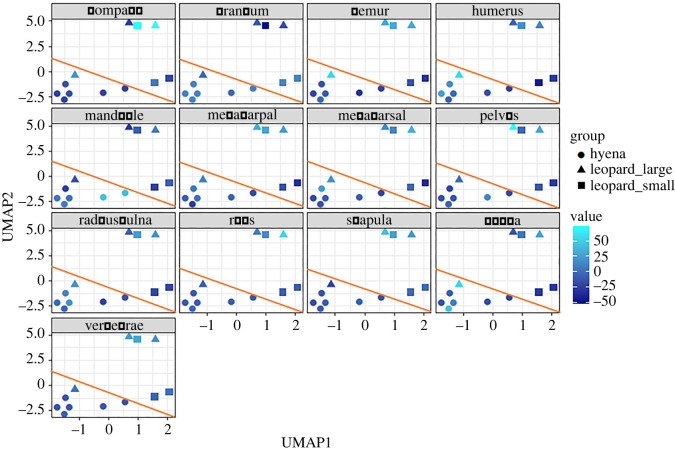
UMAP two-dimensional solution of leopard- and hyena-made bone assemblages per skeletal element. Colour indicates value intensity of each element per assemblage. The diagonal line separates cluster space of the hyena assemblages (below), and the leopard assemblages (above).

The UMAP analysis yielded similar results. The automated search grid selected an optimal model based on the use of a minimum number of three neighbours, a minimal distance of 0.1, a Manhattan distance and 50 epochs. The resulting solution separated clearly all leopard assemblages (except Hakos) regardless of carcass size. Leopard assemblages were distributed with positive values in UMAP1 and further split into two clusters in UMAP2 clearly showing differential distribution according to carcass size (figure 4). %MAU importance for each element can be seen in figure 5. There, it can be seen that cranio-mandibular elements, as well as some long bones (zygopods and metapods) are more represented in hyena-accumulated assemblages. In this model, Misiam was clearly classified within the cluster of leopard medium-sized/large carcass subsample. The Hakos medium-sized carcass subsample was the only outlier and was found within the hyena space. This could be because it is the smallest sample of leopard-modified carcasses. The paucity of axial and compact remains may also be indicative of additional attrition processes, among them the intervention of hyenas. This palimpsestic assemblage would thus have a mixed signal, strongly biased toward hyena intervention.

**Figure 6.**
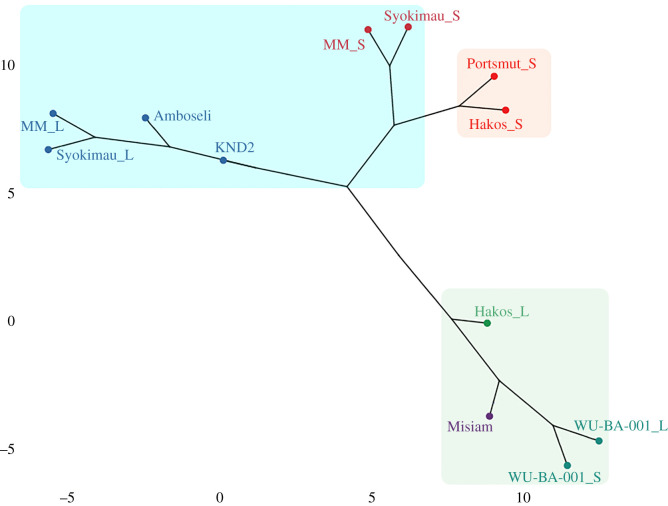
Hierarchical cluster analysis of the %MAU of hyena- and leopard-made faunal assemblages. Subsamples of small and large carcasses appear separate.

The final additional classification step, the hierarchical clustering analysis, yielded a phylogenetic tree that clearly separated all leopard assemblages from those made by hyenas (figure 6). Here, Hakos appeared clearly separated from the hyena-modified assemblages, as was also the case for the GLRM model. Small carcasses from two of the leopard assemblages (Hakos and Portsmut) were clustered close to the small-carcass hyena assemblages, but they kept their separate identity. The biggest difference documented was between the hyena and leopard larger carcass subsamples. There, Misiam was, again, clearly classified as a leopard assemblage, with limited impact by hyenas.

### 3.2. Bone breakage and element modification

**Figure 7.**
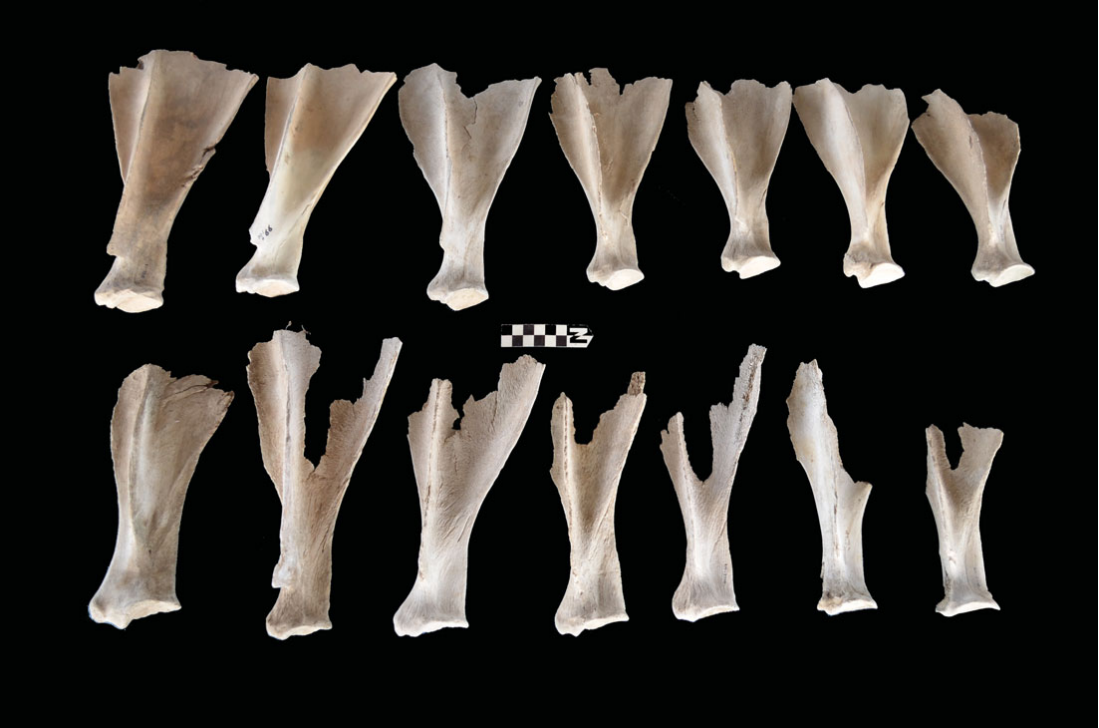
Range of variability of bone damage documented on scapulae at Misiam.

**Figure 8.**
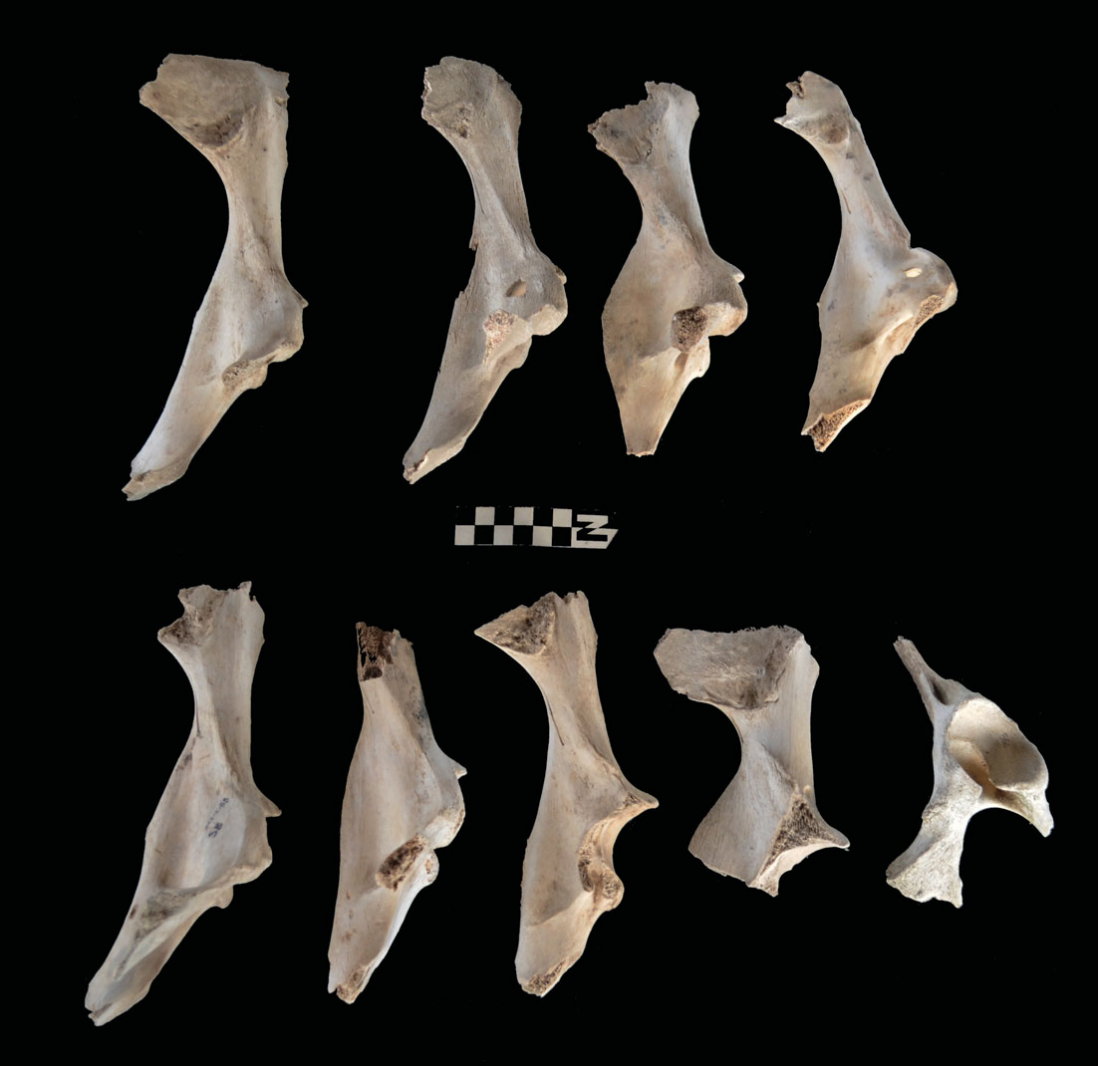
Range of variability of bone damage documented on innominate elements at Misiam.

**Figure 9.**
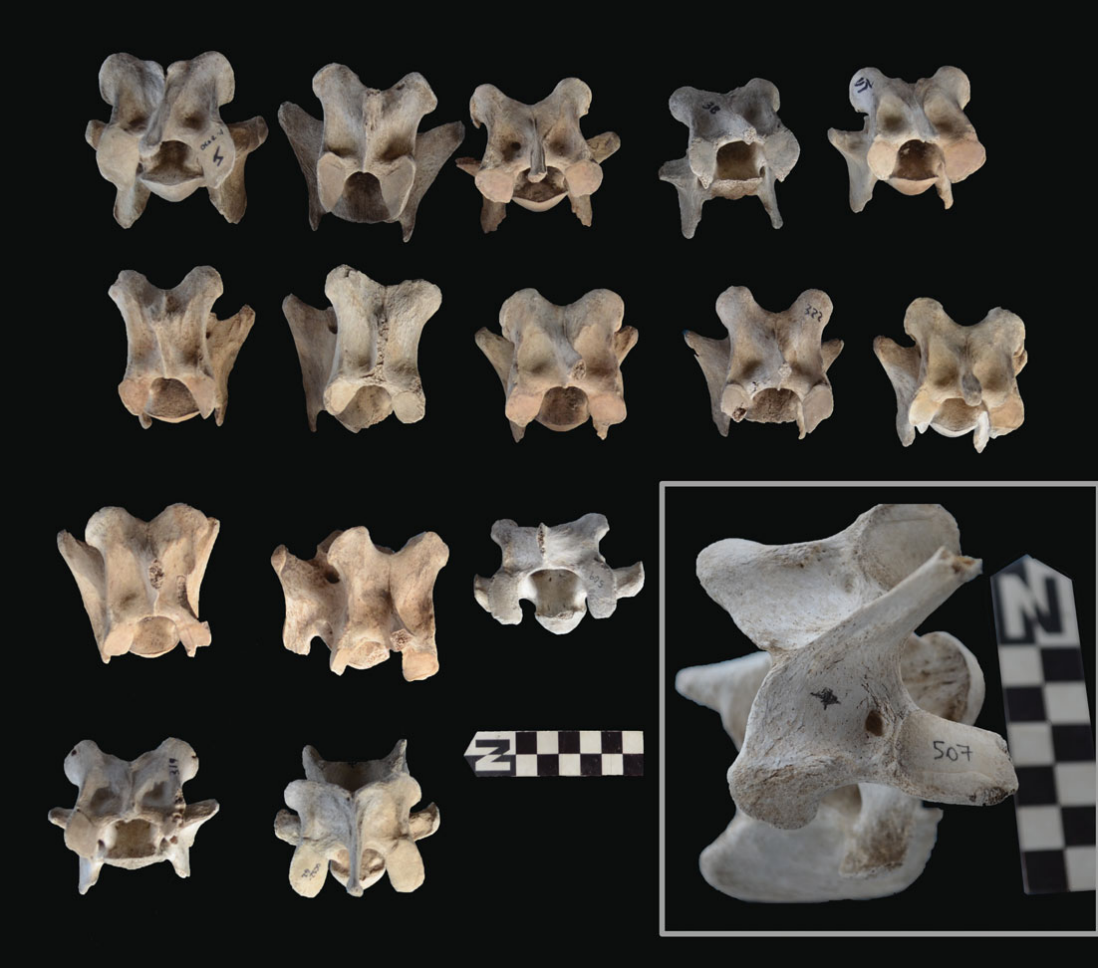
Range of variability of bone damage documented on cervical vertebrae at Misiam. Notice the furrowing on the sagittal process and the tooth pit in the inset specimen.

**Figure 10.**
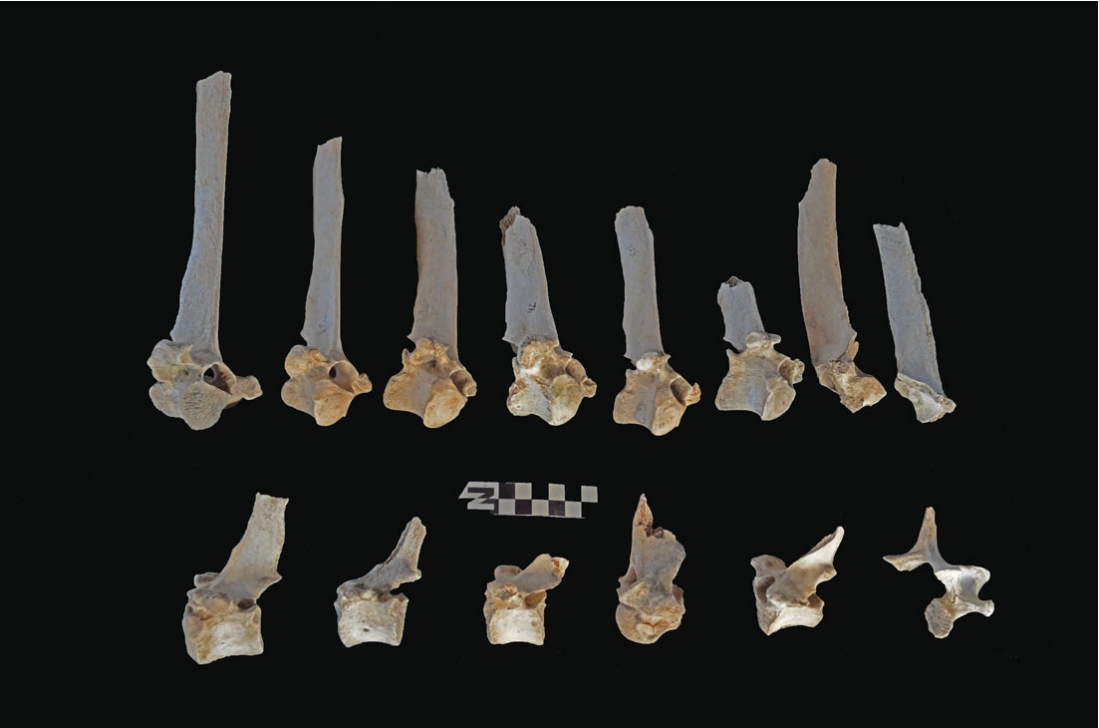
Range of variability of bone damage documented on thoracic vertebrae at Misiam.

**Figure 11.**
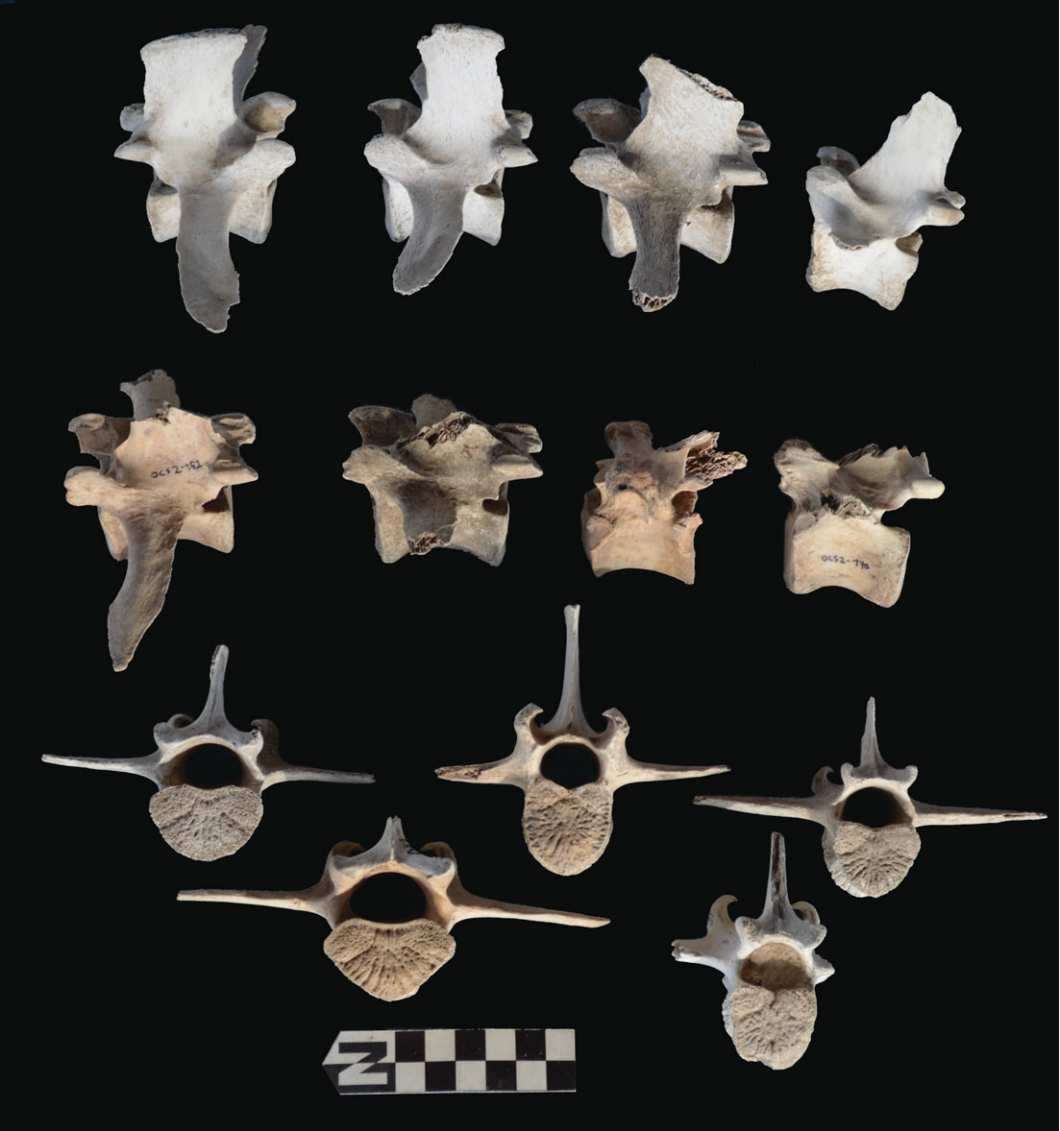
Range of variability of bone damage documented on lumbar vertebrae at Misiam; compare with thoracic vertebrae.

**Figure 12.**
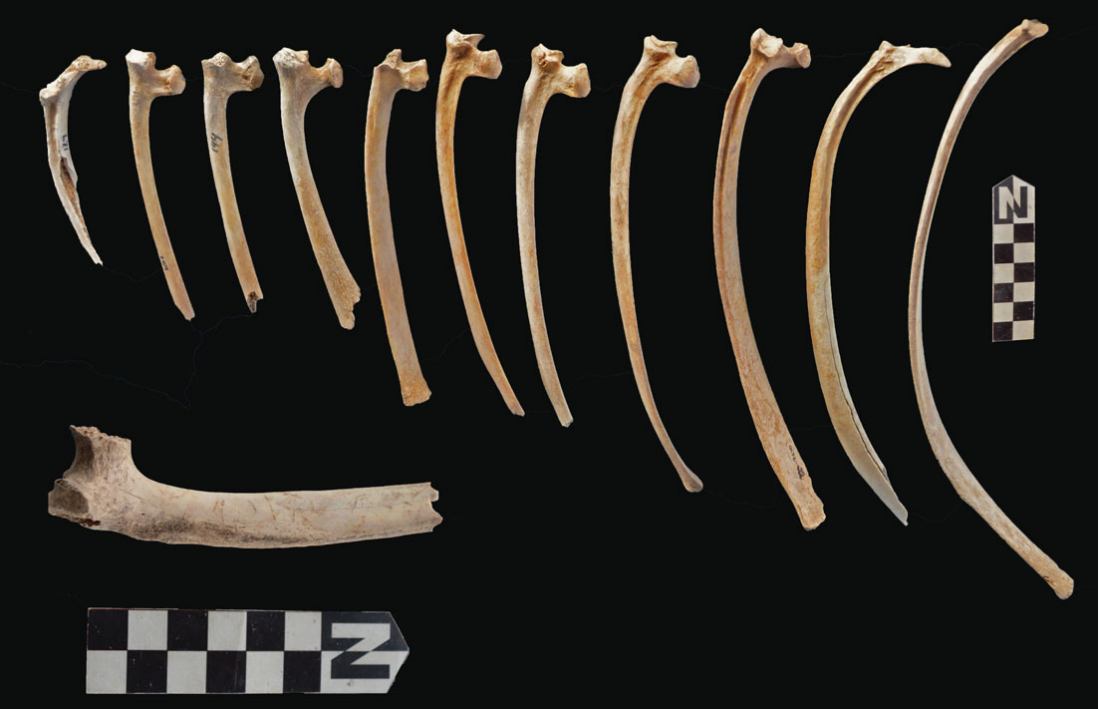
Range of variability of bone damage documented on ribs at Misiam.

**Figure 13.**
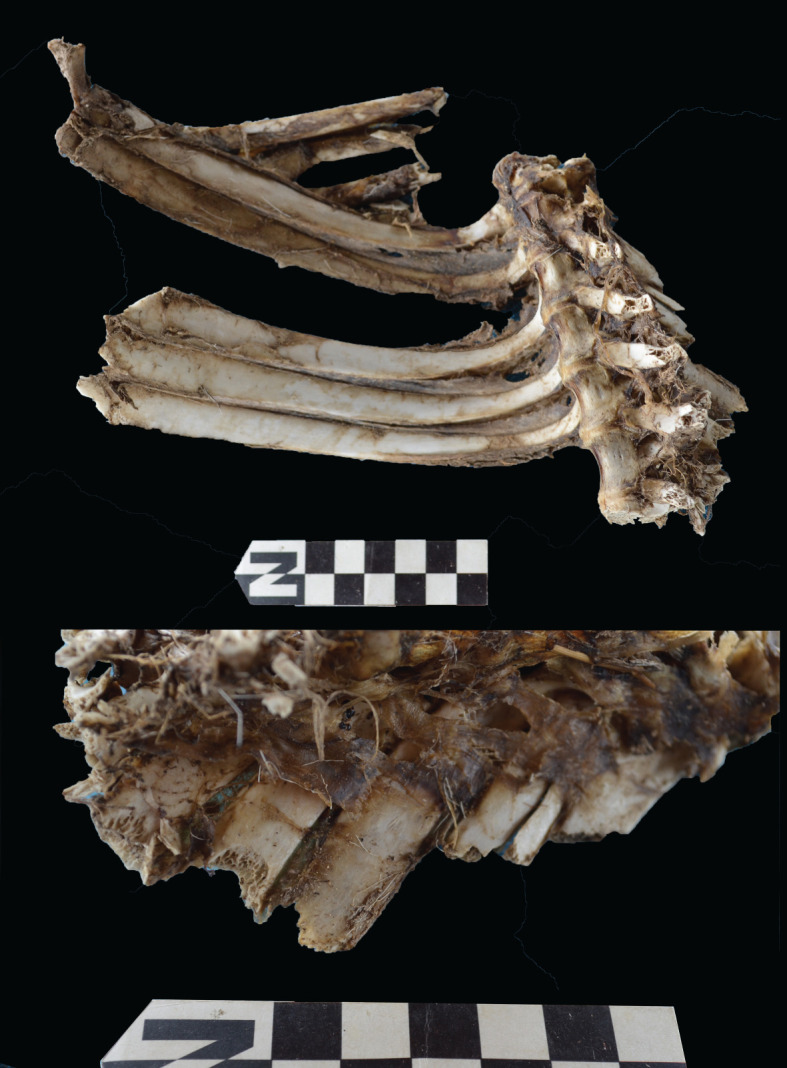
Close-up on the damage made by leopards on a gazelle axial skeleton. Notice the furrowing on the ribs and the transverse processes of thoracic vertebrae.

Scapulae appeared well preserved, with most damage concentrated on the proximal end of the blade (figure 7). The most intensive destruction was documented on the blade and the spine. The distal end was mostly undamaged with only occasional tooth-marks. Modification of the coxal elements is also moderate, with most damage concentrated on the iliac blade and the ischial portion (figure 8). The damage documented is mostly in the form of furrowing and very little tooth-marking. This trend is continued on the axial skeleton. The preserved vertebrae appear very complete, with only green breakage occurring on processes. The vertebral bodies are mostly intact and so are the neural arches. Cervical vertebrae show some limited furrowing on the spinous process (figure 9). Thoracic vertebrae also concentrate most damage on the spinous process, and to a lesser degree, on the transverse process (figure 10). This is also the pattern for lumbar vertebrae. In this case, transverse processes are more furrowed than the spinous ones (figure 11). The most conspicuous damage is documented on the spinous processes of thoracic vertebrae and ranges from marginal to almost complete deletion of the process. Damage on the vertebral bodies is rather marginal, with limited furrowing, and when existing, it is mostly in the form of isolated tooth-marks. Ribs also display a variable degree of damage, spanning from moderate furrowing of distal ends to deletion of more than two-thirds of the shaft (figures 12 and 13). Interestingly, in most ribs the articular head is preserved and unmodified. In some rib specimens, some minor furrowing is documented on the tubercles.

**Figure 14.**
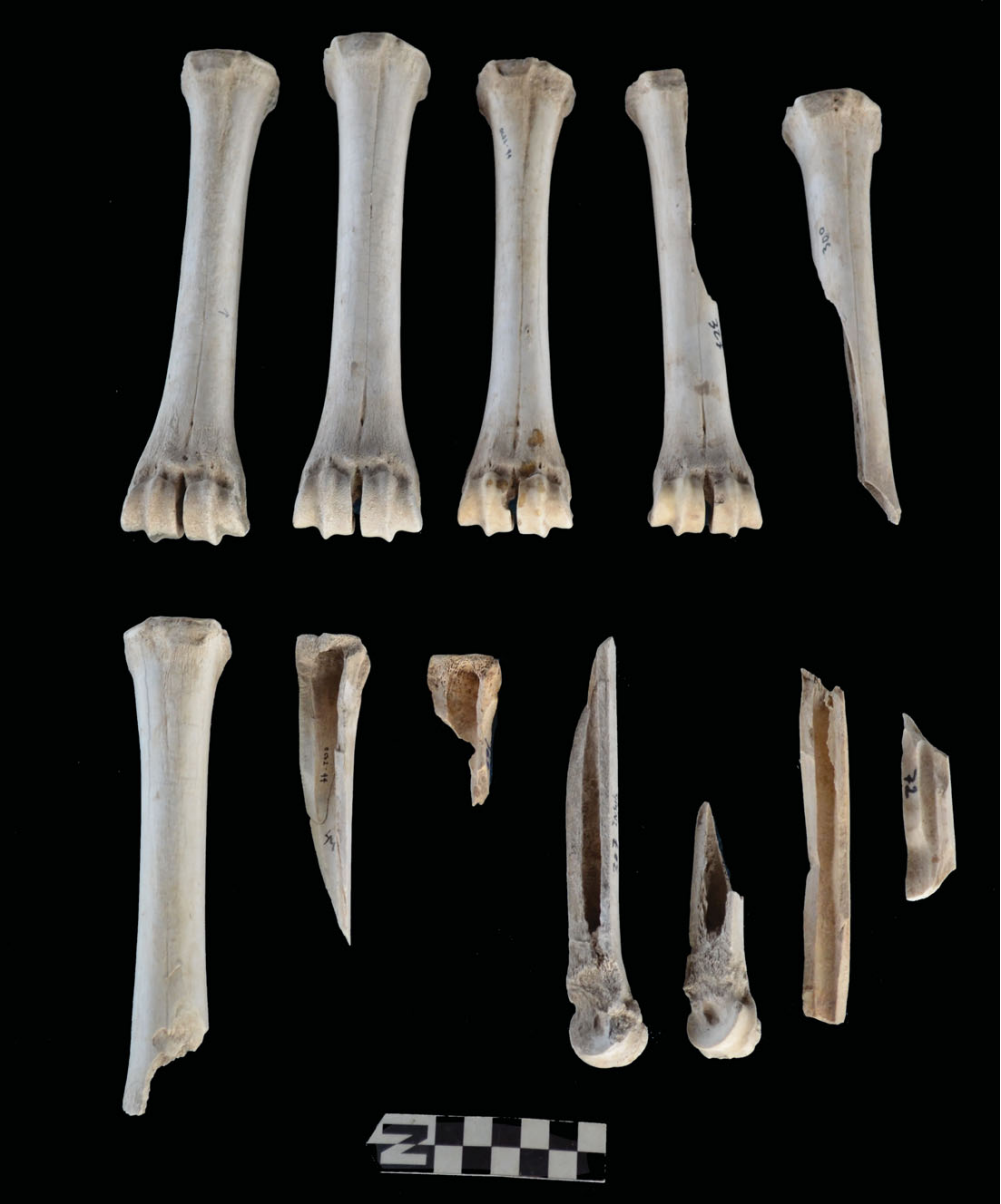
Range of variability of bone damage documented on metacarpals at Misiam.

**Figure 15.**
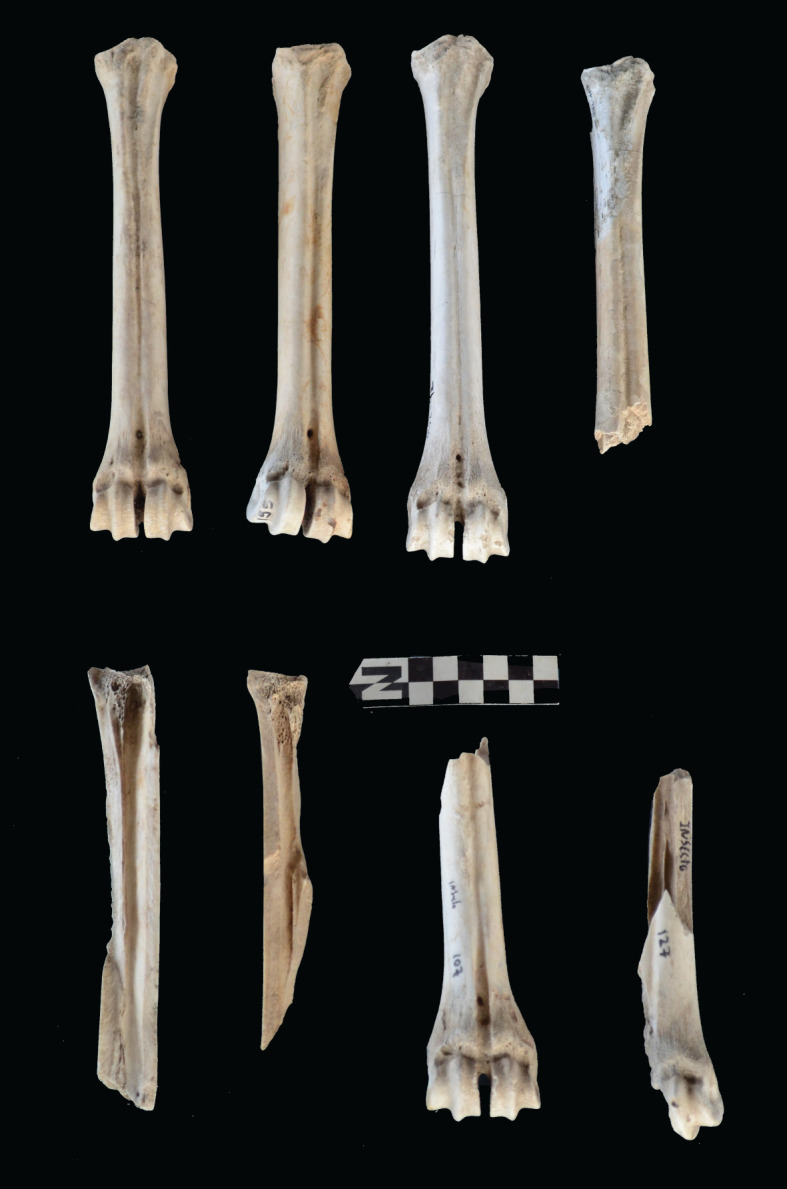
Range of variability of bone damage documented on metatarsals at Misiam.

**Figure 16.**
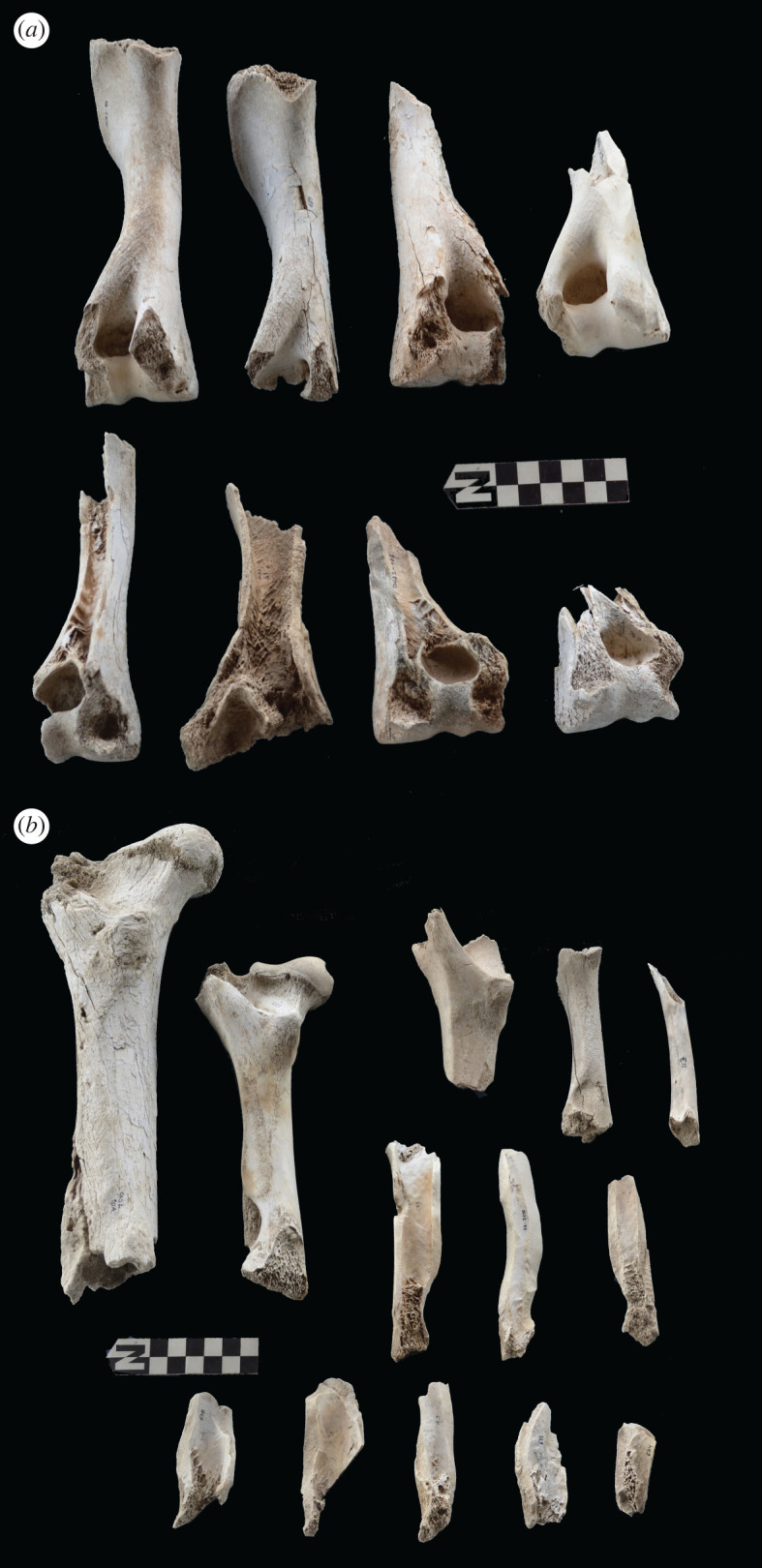
Range of variability of bone damage documented on humeri (*a*) and femora (*b*) at Misiam.

**Figure 17.**
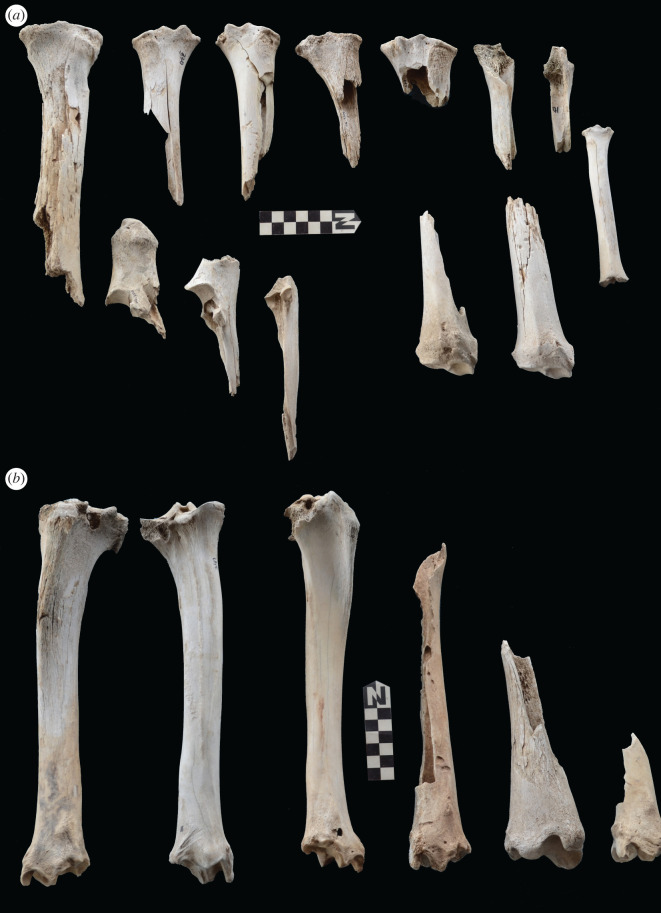
Range of variability of bone damage documented on radii-ulnae (*a*) and tibiae (*b*) at Misiam.

**Figure 18.**
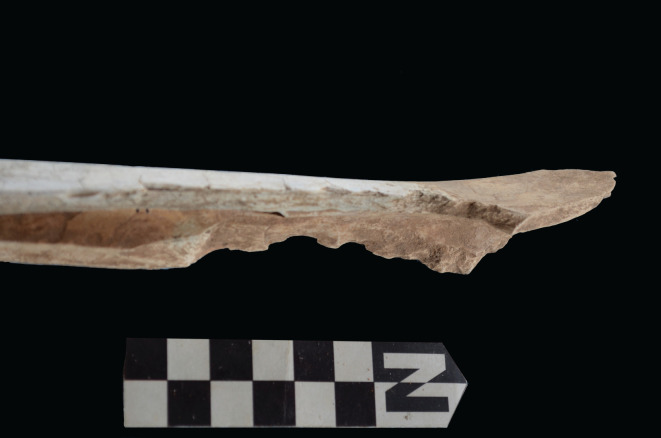
Example of multiple overlapping notch series on a tibial specimen from the Misiam assemblage.

The intervention of hyenas could be indirectly inferred only from the deletion of axial specimens, but not from any of the damage patterns documented on the preserved axial skeleton. This is not the case for long bones. Felids are known for leaving most metapodials intact, especially for size 3 carcasses [52,53]. At Misiam, both metacarpals and metatarsals range from being complete and undamaged to being highly fragmented (figures 14 and 15). All the fragmentation documented is in the form of green breakage planes, and caused by static loading by carnivores. These planes are often accompanied by notches. This observation is also documented on stylopods and zygopods. Femora are all modified (figure 16*b*). The damage documented ranges from furrowing of trochanters and distal ends, to complete deletion of epiphyseal ends and intense fragmentation, with MNE estimates derived better on shafts preserving landmarks. There are only a couple of cylinders, but from immature individuals. This could be the impact of leopards rather than hyenas, since for such small animals, hyenas would most likely have destroyed the complete bone. The intense fragmentation of the adult femora, accompanied by tooth-marking, is suggestive of hyena agency [46]. The same process is documented on tibial elements (figure 17*b*). There is a bias of preservation of distal ends. Although some tibial specimens have been preserved undamaged, in others the proximal ends have been furrowed away. In a few cases, there are some type C notches showing as many as four overlapping notches, indicative of hyenid rather than felid agency (figure 18) [54]. Large tooth pits associated with green breakage planes further support this interpretation, given the substantially smaller tooth size of leopards [55]. There is, nevertheless, a sample of tibial elements that are complete and display minor furrowing damage only on the tibial crest or some tooth-marking on the rim of the proximal articular surface. This is typical of felids [14–16]. These specimens have also escaped the attention of hyenas.

Damage on humeri is concentrated on the proximal epiphysis (figure 16*a*). Distal epiphyses are also impacted by carnivore modification, especially on both caudal epicondyles. A similar degree of damage is documented on radii-ulnae (figure 17*a*). Only one radius-ulna is preserved completely. In some elements both epiphyses have been deleted and only shafts survive. This contrasts with the felid typical pattern, in which radii are abandoned unmodified and most damage is documented only on the ulnar olecranon [14,17,46,52,53,56–58]. There is, importantly, a virtual absence of gnawing (i.e. the occurrence of multiple tooth-marks on the same bone specimen), which is typical of bone damage documented at hyena dens (see below) [46]. Given the overall complete state in which leopards abandon medium-sized and large carcasses upon consumption [12], it is remarkable that in the Misiam wildebeest assemblage, no humerus, only one femur (4.7%), three radius-ulna (27.2%), four tibiae (33.3%), 11 metacarpals (50%) and six metatarsals (33.3%) have been preserved complete. This implies a substantial degree of bone destruction, which is most likely the result of hyenid intervention. This intense modification of long bones by hyenas probably indicates that a substantial part of the axial skeleton and compact bone missing could also be the result of their intervention and not sampling bias.

**Figure 19.**
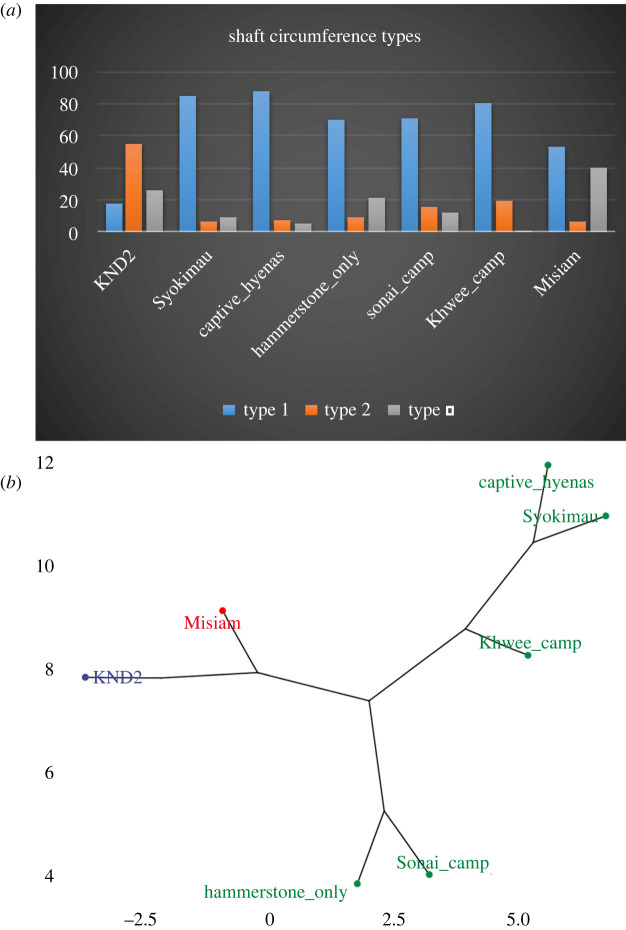
(*a*) Frequency distribution of long bone circumference shaft types. (*b*) Phylogenetic hierarchical cluster analysis of the comparative samples and the Misiam assemblage. Label colour indicates grouping when *k* = 3. Notice that distance between Misiam and KND2 and the rest of the assemblages is bigger than when comparing all adult hyena and human assemblages among themselves.

Long bone shaft circumference distribution has also been argued to indicate the type of bone modifying agency and the degree of hominin-carnivore interaction [21,32]. Unfortunately, the carnivore experimental dataset for this variable has focused exclusively on hyenas. The generic value of the term ‘carnivore’ must be questioned, since different types of carnivores may yield different shaft profiles. This is the case with Misiam, representing a leopard-accumulated assemblage (table 4). The comparison with the modern hyena and human assemblages shows that it differs substantially from both analogues. A *k* = 3 phylogenetic hierarchical cluster analysis shows that there is pronounced variance within the hyena and human groups (figure 19). Although assemblages impacted by adult hyenas are very similar, they differ from breeding dens (KND2) where immature animals are the main bone modifiers. This latter occurs in its own space, in proximity with Misiam, but clearly separated from it by its lower type 1 shafts and its higher type 2 shaft types. Human assemblages also exhibit wide variation, but they differ from hyena assemblages by their lower presence of type 3 shafts. The phylogenetic distance analysis shows KND2 sufficiently different from the rest so as to be classified by its own group (figure 19). The Misiam assemblage is also identified as its own variance group. In comparison, within the wide Euclidean space, the other assemblages show less inter-assemblage variance, so as to be classified within a single group.

**Table 4.** Long limb bone distribution: number of identified specimens, number of complete elements, epiphyseal portion representation, shafts and shaft circumference type distribution per element from the Misiam assemblage.

	NISP	complete	epiphyses^a^		shafts	shaft circumference type		
			prox	dist	*N*	type 1	type 2	type 3
humerus	24	0	0	6	18	17	1	6
radius	27	3	6	2	16	14	5	8
ulnae	5	0	3	0	2	0	0	5
metacarpal	28	11	6	5	6	7	1	20
femur	28	1	6	0	21	21	1	6
tibia	35	4	0	3	28	23	1	11
metatarsal	21	6	6	4	5	8	1	12
TOTAL	168	25	27	20	97	90	10	68

^a^It does not include the epiphyses of complete bones.

**Figure 20.**
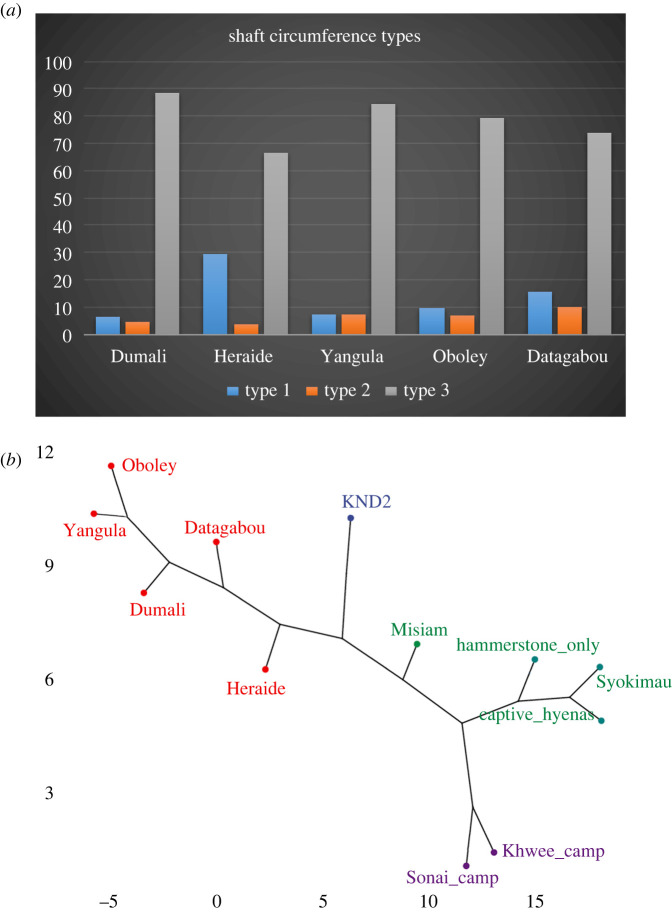
(*a*) Frequency distribution of long bone circumference shaft types from the additional hyena dens with small carcasses. (*b*) Phylogenetic hierarchical cluster analysis of the comparative samples and the Misiam assemblage. Label colour indicates grouping when *k* = 5. Notice that distance between Misiam and the rest of the assemblages is bigger than among the rest.

This is also observed when the additional spotted and striped hyena dens are added to the analysis. These additional assemblages are mostly composed of small fauna (ovicaprids), and they are a good addition for exploring variability ranges in the form in which different animal sizes are consumed at different types of hyena dens. The preservation of axial remains as well as the minor breakage undergone by these additional assemblages suggest that those dens acted as breeding dens for hyenas, and most damage was probably made by juvenile individuals, as in KND2 [31]. This is especially shown in the predominance of complete long bone shaft sections (figure 20). The Euclidean distances among samples clearly separate the anthropogenic assemblages and the medium-sized carcass hyena dens (probably consumed by adult individuals) on one end, and the small-carcass hyena dens on the other end. Misiam, again, has its own space, in between these groupings.

The shaft analysis indicates that the much more limited breakage of the Misiam assemblage is translated into a high presence of type 3 shafts and very few type 2 shafts. This is the opposite pattern to that documented in KND2. Given that Misiam contains a mixed taphonomic signal of leopards and hyenas, this indicates that leopard assemblages would typically be dominated by shafts displaying a complete section (type 3). Shaft circumference distribution also shows that the assemblage was not made by hyenas, and has limited evidence of its impact. This indicates that the generic ‘carnivore’ label should be disused and that carnivore-specific labels should be used instead. The analysis also shows that despite variability, patterns specific of agents (i.e. human, hyenas and leopards) and specific of types of dens (dominated by small or large faunas, and consumed by adult or immature individuals) exist.

Only 19 specimens bearing notches have been documented. Most are single complete notches (63.1%), followed by single incomplete notches (15.7%), overlapping notches (15.7%) and double-opposing notches (5.2%). In the only hyena assemblage (Maasai Mara hyena den), where notch types had been measured and quantified [54], overlapping notches (ratio = >0.4) and double-opposing notches (ratio = >1) were very abundant compared with single notches. This is not the case at Misiam, where type C : type A notch ratio is 0.25 and type D : type A notch ratio is 0.08.

### 3.3. Bone surface modifications (BSM)

Only 183 (32.1%) of the 569 bone specimens bear tooth-mark modifications in the form of pits, scores or furrowing (table 5). This proportion holds also when only long bone specimens are tallied (33.1%). This is lower than documented in several modern spotted hyena dens, especially when inconspicuous tooth-marks are tallied [46]. With the exception of the atlas, tooth-mark frequency on the vertebrae range from 16% to 40%, mostly in the form of furrowing of processes. Scapulae (71%) and coxal (66%) elements are highly tooth-marked. Tooth-marked long bone specimens range from 25% (radius) to 42% (tibia). Metapodials appear also as highly tooth-marked, with metacarpals showing as many as 35% of their specimens bearing at least one tooth-mark. Given the breakage patterns documented, we assume that a substantial amount of tooth-marking on limb bones was imparted by hyenas. This indicates that the original tooth-mark frequency created by leopards is substantially lower.

**Table 5.** Number of identified specimens and of tooth-marked identified specimens in the Misiam assemblage. Numbers in parentheses indicate tooth-mark percentages.

	NISP	TM-NISP
cranium	16	0(0)
hemimandible	10	1(10)
atlas	6	0(0)
axis	12	2(16.6)
vtb. cerv.	38	13(34.2)
vtb. thor	92	27(29.3)
vtb. lumb	25	10(40)
ribs	132	29(21.9)
sacrum	3	1(33.3)
coxal	39	26(66.6)
scapula	28	20(71.4)
humerus	24	10(41.6)
radius-ulna	27	7(25.9)
metacarpal	28	10(35.7)
femur	28	8(28.5)
tibia	35	15(42.8)
metatarsal	21	4(19)
compact bones	5	0(0)

Tooth-marks are not the only modification identified in the Misiam assemblage (table 6). Marks made by insects, namely beetle larvae, are also very common. About 27% of bone specimens bear evidence thereof. They are more abundant in the grease-bearing cancellous elements of the axial skeleton. Biochemical marks caused by the symbiotic action of plant roots and fungi are documented on 35% of all bone specimens.

**Table 6.** Number of specimens bearing insect-made marks, biochemical marks and weathering stages of all skeletal elements at Misiam.

	NISP	insect marks	biochemical marks	weathering stages							
				0	1	2	2–3	3	3–4	4	5
hemimandible	10	1	2	4	2			1			
vertebrae	173	38	32	93	38	28	7	5		1	1
ribs	132	47	73	67	32	25	3	3		2	
sacrum	3	1					1				
pelvis	39	14	7	11	10	6	4	6		2	
scapulae	28	11	13	3	3	8	3	5	2		4
humerus	24	3	3	12	4	1	2	3	1	1	
radius-ulna	27	13	12	14	6	4		1	1	1	
metacarpal	28	5	17	13	13	1		1			
femur	28	3	12	26		1	1				
tibia	35	6	15	18	9	4	1	2		1	
metatarsal	21	10	10	12	2	4	2	1			
compact bones	5	1	2	2	3						
total	566	153	198	235	109	82	24	28	4	8	5
%NISP		27.0	35.0	41.5	19.3	14.5	4.2	4.9	0.7	1.4	0.9

### 3.4. Subaerial weathering

The Misiam faunal collection exhibits different types of subaerial weathering modifications. Using Behrensmeyer's weathering stages, most of the assemblage displays stages 0–1 (greater than 60%) (table 6). Stage 2 reaches 14.5% of the assemblage. Only one quarter of the collection displays higher (stages > 2) weathering modifications. However, these estimates are dependent on skeletal part representation. Axial elements seem to weather faster than elements with dense cortical bones. If we focus the analysis on just long limb bones, weathering is very light: stage 0 (58.2%), stage 1 (20.8%), stage 2 (9.2), stage > 2 (11.6%) (table 6). If we take Behrensmeyer's estimated rates of weathering, this would imply that a minimum of 41% (all skeletal elements) and a maximum of 58% (just long limb bones) of the assemblage was accumulated in less than a year. More broadly, if combining stages 0 and 1, this would imply that minimum of 60% (all skeletal elements) and a maximum of 79% (just long limb bones) of the assemblage accumulated between 0 and 3 years.

### 3.5. Orientation patterns

The Rayleigh test (*K* = 0.06, *p* = 0.593), the Kuiper test (*V* = 1.35, *p* > 0.15), and the Watson's test (U^2^ = 0.069, *p* > 0.10) of the subsample analysed yielded an isotropic distribution, showing that the movements of bones along the slope were either limited or unaffected by their downward transportation by the effect of gravity, soil movement and rains. The lack of anisotropy could also be due to the test pit being an original deposition locus, since the bones collected showed an anomalous density compared with the surrounding area and included skeletal parts of various individuals.

## 4. Discussion

Despite more than half a century of actualistic work on modern carnivore bone accumulations, there is still a paucity of assemblages for all bone-accumulating carnivore agents, especially from those in Africa. Less than a dozen spotted, brown and striped hyena dens with a representative sample of bones have been taphonomically studied, especially with a diverse set of taphonomic variables involving more than just anatomical profiles and frequency of tooth-marks [45,59–61]. This is probably insufficient to address the issue of behavioural variability of these agents. The situation is worse for most other carnivore taxa. Only one accumulation has been interpreted as lion-made [2], and less than six leopard-made accumulations have been taphonomically studied [1,9,10,12,13]. This is also insufficient to determine the range of behavioural variability by these felids. Therefore, more actualistic assemblages should be studied within the ecological contexts in which they were generated. The analysis of the Misiam assemblage intends to increase our knowledge on wild felid (namely, leopard) bone accumulations. It also intends to show that although single-agent processes are probably documented in many hyena dens, this is not the case for leopard-made assemblages. Intervention of scavenging agents is the rule rather than the exception. Brain [1,9,10,12,13] argued that hyenas, small canids and porcupines probably intervened in the leopard lairs that he documented. At Misiam, we document the intervention of hyenas by the biased representation of the axial skeleton, the intense fragmentation of a part of the long limb bone sub-assemblage, the presence of fragmented metapodials and the presence of large tooth-marks on bones, which contrast with the small size of leopard tooth-marks [55,62]. The lack of taxonomic diversity, carnivore remains, coprolites and digested bone, all common in hyena dens, indicate that the role of hyenas at Misiam was of post-depositional ravaging and not as an accumulating agent. The damage patterns documented, especially on the axial skeleton and on the complete long bones, are typical of felids. The physical presence of leopards at the site reinforces the interpretation.

Misiam is most similar to previously documented leopard-accumulated assemblages, where medium-sized carcasses are predominant (WUBA001). The skeletal profile and gross bone damage documented at Misiam is also similar to the medium-sized carcass portion of other assemblages where medium-sized carcasses are not so predominant (e.g. Hakos) (figure 3). This is broadly summarized in the higher presence of the low-survival skeletal set (ribs, vertebrae, pelvis and compact bones) in these assemblages compared with others where hyenas have had a major impact or where they are the predominant accumulating agent (figure 3). This is reflected to a lesser extent in the bone surface modification frequencies. At Misiam, the tooth-mark frequencies reported are higher than those documented in other assemblages where hyenid impact is minor or non-existent. For example, at OCS (a felid-accumulated assemblage with very low scavenging impact) a total of 7% of elements are tooth-marked [2]. This is in accordance with the tooth-mark frequencies reported for modern exclusively felid-modified assemblages [53,57,58]. The higher percentage of tooth-marks at Misiam attests to a higher impact of hyenid post-depositional intervention. This would also explain the substantial deletion of some of the low-survival element set, especially of vertebrae, ribs (under-represented if considering MNI) and compact bones.

Behrensmeyer reports that subaerial weathering stages 3 and up can occur at any time between 6 years and more than 15 years of exposure. She also documents that in no case any of the carcasses that she monitored exposed longer than 3 years showed stages 0 or 1. This is documented regardless of habitat. Report of carnivore use of Misiam has been documented for the past 13 years. Most likely, it existed before, but it was not called to our attention by the local Olduvai Maasai population. We were only able to collect the bones after the vegetation was cleared in 2016. We have monitored the site for 4 years, but only intermittently because of interruption of work at the gorge caused by the pandemic global situation. We know that during this time there was a deposition of a minimum of four carcasses. Whereas it is true that these carcasses were mostly at stage 0 or 1, we also documented some differences according to elements. In one carcass, most stage 1 was observed on axial bones. What we know for sure is that the bulk of the assemblage was not deposited over a maximum span of 3 years, as suggested by the predominant weathering stages. The presence of several elements in stages 4 and 5 suggests that there were carcasses deposited over more than 15 years. However, carcasses have been brought into the area probably over most of that time span. This is not reflected in the predominance of stages 0 and 1, and this could reflect slower weathering rates inside a highly closed-vegetation area, with no exposure to sunlight. Behrensmeyer remarked that there were more bones reflecting stages 0 and 1 in certain habitats, among which dense woodland was one of them. She said that a smaller contrast between diurnal–nocturnal temperatures, a higher moisture and less direct sunlight could slow down dehydration and weathering of bones in these environments. It could be argued that Misiam is sampling carcass deposition behaviours for at least 15 years, probably with variable rates along this period. The predominance of slight weathering stages would suggest that the bulk of the carcasses may have been deposited over the past 10 years.

Sites like Misiam seem to be common at Olduvai Gorge. In steep crevices and ravines where dense vegetation concentrate, leopards find bush and tree patches that are ideal for their discrete behaviour. The two that we have documented, with leopards in them, are equally situated in the uppermost section of the gorge walls, right close to the rim of the gorge, in the vicinity of the short-grass plains that are covered with wild game during the rains. Leopards, as local predators, hunt the surrounding game to these locations; however, they do not seem to bring all their prey to their lairs. The bulk of the animals uncovered at Misiam are wildebeests. These are extremely seasonal on these plains, and are documented only during the rainy season. This indicates that even if the use of Misiam by leopards were continuous, their carcass-accumulating behaviour has a distinctive seasonal basis there. Specialized wildebeest hunting during the wet season, also supported by the presence of fetal and newly born remains at Misiam, shows that leopards can also be successful hunters of medium-sized prey. It could be argued that carcass transport, as an energy investment, is conditioned by resource competition. The higher incidence in carcass transport by leopards during the wet season could be a reflection of higher rates of competition caused by an increase in the density of spotted hyenas on the short-grass plains during this time of the year (personal observation). Leopards can manage competition from single striped hyenas, the most commonly documented hyenas in Olduvai Gorge, but they cannot do the same with the larger numbers of spotted hyenas. This is what prompts their tree-stored carcass behaviour in Seronera [63], and it can be argued that this also leads them to accumulate higher rates of carcasses at Misiam and other similar lairs.

Another wildebeest-dominated assemblage discovered at Olduvai Gorge (OCS) with more than 50 wildebeests, was interpreted as an exceptional accumulation made by lions [2]. The main arguments for the interpretation were: the highly specialized taxonomic profile, the more likely attribution of a medium-sized prey to a large felid, the clear felid pattern of bone modification, and an assemblage where hyena modification was virtually non-existent. At least one skeleton was fairly complete and appeared articulated, and no more than 7% of bones were tooth-marked. The discovery of Misiam should make us revisit OCS and the issue of agency. Although the location of OCS is relatively open and very different from the type of sheltered locations that leopards seek for their lairs, one could wonder if OCS was also accumulated and modified by leopards. In the case of Misiam, we are secure of the agency because the agent has been physically documented. This underscores that leopards can also be major agents in the accumulation of medium-sized carcasses. Whether they hunt them or scavenge them taking advantage of the mass killings made by lions that exceptionally take place during the wildebeest migration in the birthing season [64], both strategies could make leopards, who usually are generalists, specialists on a seasonal basis. An alternative interpretation would be that wildebeest at Misiam were accumulated by lions, and that leopards contributed with smaller game. Against this interpretation, the local Maasai living nearby described to us how leopards are commonly seen at Misiam, but not lions. This was also confirmed by us, since in the moment in which we collected the newly deposited carcasses in 2022, two leopards were documented at the place. Likewise, a second leopard lair that we documented by the fifth fault contained a large leopard male, which made it impossible to retrieve all the bones that we saw there, but the few elements that we collected belonged to three different wildebeests. Therefore, wildebeest are targeted by the leopards in the gorge. This is not new. Wildebeest remains were found in the leopard lair documented by de Ruiter and Berger [12]. They were accumulated together with even larger game, including waterbuck, kudu, buffalo, Burchell's zebra and even eland by a leopard that was monitored for a few years. The fact that leopards can obtain carcasses of those sizes and drag them from the kill to the cave, even taking them to the innermost recesses, is informative. Red river hogs (weighing as much as 285 pounds) and forest buffalo (weighing 550–700 pounds) are the two main prey of leopards in the Lopé National Park (Gabon) [65]. Therefore, the assumption that medium-sized and larger prey must be hunted by lions should be nuanced in light of the wide range of carcass sizes preyed upon and consumed by leopards.

It is certainly a fascinating coincidence that these types of behaviours and taphonomic agencies can also be traced back in time next to Misiam at FLK North [15] (figure 1). There, it has been inferred that felids, namely leopards or medium-sized sabre-tooth felids, were specialized accumulating agents of *Parmularius* and *Antidorcas,* among other less represented taxa. At the time, the location was ecologically very different: situated on dry ground and surrounded by wetland. Then, on an intermittent basis, hyenas had access to some of the carcasses, further modifying them. This type of felid–hyenid interaction has probably shaped the behaviour of leopards in the past few million years and continues to do so in the present. The neo-taphonomic data from Misiam can, thus, enhance our interpretation of sites like FLK North, where similar processes seem to have been at play.

Although there are substantial anatomical and adaptive differences between sabre-tooth felids and modern felids, it has recently been shown that despite their different dentitions, their carcass consumption behaviour produced very similar modification patterns [66]. For instance, the taphonomic analysis of a den of the homotherine *Xenosmilus hodsonae* showed a highly specialized prey profile (*Platygonus vetus*), with carcasses substantially modified during defleshing (including some compelling evidence of durophagy) [66]. The extensive defleshing documented on these carcasses led to destruction of the scapular blade, with occasional tooth-marking of the neck. Humeri displayed furrowing on their proximal ends and on the caudal aspect of the epicondyles (with emphasis on the medial epicondyle). In comparison, minimal damage is documented on radii, in contrast with the ulnar olecrana, which appear more extensively modified by furrowing. Femora appear furrowed on both epiphyseal sections. The proximal epiphyses of tibiae appear also impacted, with the crest showing frequent furrowing and even complete deletion. Gnawing is also documented on the iliac crest of innominates and sacral vertebrae. Lumbar and thoracic vertebrae also display furrowing on their apophyses and spinal processes. Damage on ribs is documented mostly on their distal ends. All this is virtually non-differentiable from the anatomical damage patterns inflicted by modern felids like lions, leopards or tigers [53,57,58]. This also indicates that homotherine sabre-tooth felids not only defleshed carcasses as efficiently as modern felids, but that in the process, they used their dentition in a similar manner, resulting in broadly similar modification patterns on their prey, which are more typical of felid carnivores in general. This makes their differentiation from other felids in the attribution of agency in ungulate accumulations in the fossil record more challenging. Their distinctive incisal tooth morphology and the resulting tooth-mark shapes may contribute to elucidate their role in prehistoric faunal accumulations [66].

The overlapping use of the space at Misiam by leopards and hyenas has also additional relevance for potential common-amenity scenarios of archaeofaunal assemblages. At FLK North, fauna and lithics are retrieved in the same contexts, despite the virtual lack of faunal exploitation by hominins and the taphonomic documentation of felid–hyenid interactions in the modification of carcasses [15]. This supports the interpretation of functionally independent deposition of lithic artefacts and bones at the site and provides a good example of common-amenity processes with multiple agencies. The predominant stone tool battering activities at the site [67] do not seem to have been aimed at bone demarrowing given the virtual inexistence of percussion marks, typical angular breakage planes and associated notches and bone impact flakes. This, in addition with the high percentage of complete surviving long bone elements indicates that battering activities by hominins were aimed at exploiting additional non-faunal resources, which remain unknown.

## 5. Conclusion

Misiam is a wildebeest-dominated assemblage accumulated by the action of leopards and with a double felid–hyenid agency in its modification. The assemblage shows the specialized nature of the accumulating agent, despite its impact in a wider range of fauna. The specialization seems artificial, since it focuses mostly on one taxon that is only available during the wet season, despite the eclectic dietary habits that characterize leopards all year round [68,69]. Either competition or breeding modulate the intensity of carcass transport and accumulation at the site. Hyenas have intensively modified the assemblage, most likely upon carcass discard by leopards. Misiam, thus, constitutes a good neo-taphonomic example of a palimpsestic carnivore-made carcass accumulation with inter-taxonomic interaction. Given that most felid-made (namely, leopard) accumulations will be impacted to different degrees by scavengers, Misiam offers a good analogue for the taphonomic identity of such interactions. It also offers a good analogue for palaeontological and archaeological assemblages where felid and hyena agencies can be detected. Future research should target more leopard lairs along the gorge to identify variability in their behaviour and in the impact by hyenas, and to test whether accumulation rates are also specialized and seasonally dependent.

## Data Availability

Data is included in the manuscript. The raw data (tables MAU, MAU leo, Sites) as well as the code (glrm) are included as electronic supplementary material, files [70].
